# Image-based quantitative analysis of tear film lipid layer thickness for meibomian gland evaluation

**DOI:** 10.1186/s12938-017-0426-8

**Published:** 2017-11-23

**Authors:** Hyeonha Hwang, Hee-Jae Jeon, Kin Choong Yow, Ho Sik Hwang, EuiHeon Chung

**Affiliations:** 1 Department of Electrical Engineering and Computer Science, Gwangju Institute of Science and Technology (GIST), 123 Cheomdan-gwagiro, Buk-gu, Gwangju, 61005 Korea; 2 Department of Biomedical Science and Engineering, Institute of Integrated Technology, Gwangju Institute of Science and Technology (GIST), 123 Cheomdan-gwagiro, Buk-gu, Gwangju, 61005 Korea; 3 Department of Ophthalmology, Chuncheon Sacred Heart Hospital, Hallym University, 77 Sakjuro, Chuncheon, Gangwon-do 24253 Republic of Korea; 4 School of Mechanical Engineering, Gwangju Institute of Science and Technology (GIST), 123 Cheomdan-gwagiro, Buk-gu, Gwangju, 61005 Korea

**Keywords:** Dry eye syndrome, Lipid layer thickness, White light thin film interference, Color compensation, ROI (region of interest) tracking, Meibomian gland dysfunction

## Abstract

**Background:**

Dry eye syndrome is one of the most common ocular diseases, and meibomian gland dysfunction (MGD) is the leading cause of evaporative dry eye syndrome. When the tear film lipid layer becomes thin due to obstructive or hyposecretory meibomian gland dysfunction, the excessive evaporation of the aqueous layer can occur, and this causes evaporative dry eye syndrome. Thus, measuring the lipid layer thickness (LLT) is essential for accurate diagnosis and proper treatment of evaporative dry eye syndrome.

**Methods:**

We used a white LED panel with a slit lamp microscope to obtain videos of the lipid layer interference patterns on the cornea. To quantitatively analyze the LLT from interference colors, we developed a novel algorithm that can automatically perform the following processes on an image frame: determining the radius of the iris, locating the center of the pupil, defining region of interest (ROI), tracking the ROI, compensating for the color of iris and illumination, and producing comprehensive analysis output. A group of dry eye syndrome patients with hyposecretory MGD, dry eye syndrome without MGD, hypersecretory MGD, and healthy volunteers were recruited. Their LLTs were analyzed and statistical information—mean and standard deviation, the relative frequency of LLT at each time point, and graphical LLT visualization—were produced.

**Results:**

Using our algorithm, we processed the lipid layer interference pattern and automatically analyzed the LLT distribution of images from patients. The LLT of hyposecretory MGD was thinner (45.2 ± 11.6 nm) than that of dry eye syndrome without MGD (69.0 ± 9.4 nm) and healthy volunteers (68.3 ± 13.7 nm) while the LLT of hypersecretory MGD was thicker (93.5 ± 12.6 nm) than that of dry eye syndrome without MGD. Patients’ LLTs were statistically analyzed over time, visualized with 3D surface plots, and displayed using 3D scatter plots of image pixel data for comprehensive assessment.

**Conclusions:**

We developed an image-based algorithm for quantitative measurement as well as statistical analysis of the LLT despite fluctuation and eye movement. This pilot study demonstrates that the quantitative LLT analysis of patients is consistent with the functions of meibomian glands clinically evaluated by an ophthalmologist. This approach is a significant step forward in developing a fully automated instrument for evaluating dry eye syndrome and for providing proper guidance of treatment.

**Electronic supplementary material:**

The online version of this article (10.1186/s12938-017-0426-8) contains supplementary material, which is available to authorized users.

## Background

Dry eye syndrome (DES) is one of the most common ocular diseases affecting more than 5% of the US adult population [1]. Dry eye syndrome is composed of aqueous deficiency DES and evaporative-type DES. The most common cause of the evaporative-type DES is the meibomian gland dysfunction (MGD) [1–3]. In the eye, the preocular tear film comprises of mucous, aqueous, and lipid layers. The lipid layer, formed by oil (meibum) secreted from meibomian glands, prevents excessive evaporation of the aqueous layer. In obstructive or hyposecretory meibomian gland dysfunction, thinning of the lipid layer leads to excessive evaporation and develops evaporative-type DES [4, 5]. Recently, numerous studies have been conducted on the correlation between DES and the lipid layer thickness (LLT) [6–10]. Therefore, accurate measurement of LLT has become more important.

In normal condition, the LLT has been estimated and reported to be approximately 70 nm in the eye [11, 12]. The lipid layer thickness can be estimated by analyzing the color intensity patterns generated by the optical interference from multiple reflections at the interfaces of air-lipid and lipid-aqueous layers [13]. Various methods utilizing interference patterns have been used to characterize the lipid layer thickness: gooseneck light [14], bio differential microscope [15], Polaroid filters [16], monochromatic light [17], spectral-discrimination [18], and a simple interferometer made of paper tool for lipid layer evaluation [19]. Several tear interference imaging devices have also been developed, such as the DR-1 tear interference camera (Kowa Co., Nagoya, Japan) [20, 21], LipiView interferometer (TearScience Inc, Morrisville, NC) [9, 22] and Lipiscanner 1.0 (Visual Optics, Chuncheon, Korea), a cost-effective add-on to an existing slit lamp biomicroscope. So far, only the LipiView device can provide quantitative values of the lipid layer thickness.

While the DR-1 can qualitatively visualize the interference pattern of lipid layer [23], the LipiView interferometer can quantitatively measure the average lipid layer thickness. The main proprietary algorithms and high-speed computers in these systems capture the reflected color from lipid layer at a rate of approximately 14 million pixels per second to complete the evaluation. The spatially modulated light source allows the elimination of unnecessary background images and stray light. The processed output is expressed as interference color that correlates with the thickness of the lipid layer [24].

However, complicated interferometry systems are costly (Lipiview) or may provide qualitative analysis (DR-1). By utilizing a recently developed low-cost custom-made Lipiscanner 1.0 system for quantitative measurements, we developed an image-based algorithm to automatically define the region of interest (ROI) on the iris and to evaluate the thickness of the lipid layer, even with irregular pupil movements during data acquisition. (see Additional file 1: Movie, Additional file 2: Movie, Additional file 3: Movie, Additional file 4: Movie.) Also, we performed color compensation in our algorithm to obtain a precise measurement of the lipid layer thickness that is based on the Fresnel equation [25]. To validate our algorithm, we performed a feasibility analysis on the video data obtained from twenty patients and fourteen healthy volunteer who were affected by either hyposecretory MGD, dry eye syndrome without MGD, or hypersecretory MGD.

## Theory

A colorful interference pattern is caused when the light is reflected from the top and bottom boundaries of a thin film such as an oil film floating on a bubble or water. Interferences by thin films display different colors depending on the thickness of the film, from dark color caused by thinner film area to brighter color caused by thicker film area. Using Snell’s law,

1 $$\text{OPD} = \frac{{2n_{m} d}}{\cos \beta }\left( {1 - \sin^{2} \beta } \right) = 2n_{m} d\cos \beta$$

where $\text{OPD}$ means “optical path difference,” *n*_*m*_ means “refractive index of meibum,” $d$ means “lipid layer thicknesses” and $\beta$ means “refraction angle.”

The phase difference ($\Delta$) between the two interfering lights can be calculated using the wavelength of light ($\lambda$) and $\text{OPD}$ as follows:

2 $$\Delta = 2\pi \frac{{\text{OPD}}}{\lambda } .$$

Meanwhile, the intensity of light is calculated by the Fresnel equations. The Fresnel equations are a pair of equations that describe the reflectance and transmittance of a surface between two media having different refractive indices. The reflectance and the transmittance are coefficient values representing the ratio of incident light that is reflected or transmitted. According to the Fresnel equations, the ratio of the reflected and transmitted wave’s complex electric field amplitude to that of the incident wave (reflection and transmission coefficients) for *s*- and *p*-polarization are $r_{s}$, $r_{p}$, $t_{s}$ and $t_{p}$:

3 $$r_{\text{s}} = \frac{{n_{1} \cos \theta_{1} - n_{2} \cos \theta_{2} }}{{n_{1} \cos \theta_{1} + n_{2} \cos \theta_{2} }}$$

4 $$t_{\text{s}} = \frac{{2n_{1} \cos \theta_{1} }}{{n_{1} \cos \theta_{1} + n_{2} \cos \theta_{2} }}$$

5 $$r_{\text{p}} = \frac{{n_{2} \cos \theta_{1} - n_{1} \cos \theta_{2} }}{{n_{2} \cos \theta_{1} + n_{1} \cos \theta_{2} }}$$

6 $$t_{\text{p}} = \frac{{2n_{1} \cos \theta_{1} }}{{n_{1} \cos \theta_{2} + n_{2} \cos \theta_{1} }}$$

where $\theta_{1}$ and $\theta_{2}$ mean incident angle and refractive angle, $n_{1}$ and $n_{2}$ indicate refractive index of air and meibum.

Then, the reflectance and transmittance are as follows:

7 $${\mathbb{R}} = \left| r \right|^{2}$$

8 $${\mathbb{T}} = \frac{{n_{2} \cos \theta_{2} }}{{n_{1} { \cos }\theta_{1} }}\left| t \right|^{2}$$

where ${\mathbb{R}}$ and ${\mathbb{T}}$ represent the reflectance and the transmittance, respectively.

With these coefficients, all intensities of rays can be defined in a simple reflection-transmission model. In this model, we defined $R_{m}$ (*m,* a nonnegative integer) to be the ray’s wave function which is reflected $m$ times between the lipid-tear interface and the air-lipid interface. $R_{0}$ is the wave function of the ray which is reflected directly at the surface of the lipid layer. Hence,

9 $$\begin{aligned} I_{{R_{0} }} &= {\mathbb{R}}_{12} I_{{R_{i} }} \\ I_{{R_{1} }} & = {\mathbb{T}}_{12} {\mathbb{R}}_{23} {\mathbb{T}}_{21} I_{{R_{i} }} \\ I_{{R_{2} }} &= {\mathbb{T}}_{12} {\mathbb{R}}_{23} {\mathbb{R}}_{21} {\mathbb{R}}_{23} {\mathbb{T}}_{21} I_{{R_{i} }} \\ & \ldots \\ I_{{R_{m} }} &= {\mathbb{R}}_{21} {\mathbb{R}}_{23} I_{{R_{{\left( {m - 1} \right)}} }}\quad ({\text{integer }}m > 1) \end{aligned}$$

where $I_{{R_{n} }}$ (nonnegative integer $n$) is the intensity of the light $R_{n}$, and $R_{i}$ is the incident light’s wave function. ${\mathbb{R}}_{ij}$ is the coefficient of the reflectance from $i$ layer to $j$ layer (1-air, 2-lipid layer, 3-tear layer) and ${\mathbb{T}}_{ij}$ corresponds the coefficient of the transmittance.

In this model, $\text{OPD}$ is present at every new reflection-transmission process. Therefore, the phases of the rays are described as follows.

10 $$\theta_{m} = m\Delta$$

Now we have each ray’s phase and intensity. From these, the interference ray can be calculated in the complex number plane as follows.

11 $$R_{\text{INT}} = \mathop \sum \limits_{m = 0}^{\infty } \sqrt {\left| {I_{{R_{m} }} } \right|} \text{e}^{{i\theta_{m} }}$$

$I_{\text{INT}} \left( {\lambda ,d} \right)$ can be obtained from the amplitude of the wave function $R_{\text{INT}} \left( {\lambda ,d} \right)$, which is used to obtain the RGB value as follows:

12 $$\text{Red}\left( d \right) = \mathop \sum \limits_{\lambda } I_{\text{INT}} \left( {\lambda ,d} \right) \cdot R_{\text{STDOBS}} \left( \lambda \right) ,$$

13 $$\text{Green}\left( d \right) = \mathop \sum \limits_{\lambda } I_{\text{INT}} \left( {\lambda ,d} \right) \cdot G_{\text{STDOBS}} \left( \lambda \right) ,$$

14 $$\text{Blue}\left( d \right) = \mathop \sum \limits_{\lambda } I_{\text{INT}} \left( {\lambda ,d} \right) \cdot B_{\text{STDOBS}} \left( \lambda \right) .$$

Here, $I_{\text{INT}} \left( {\lambda ,d} \right)$ is the intensity of interference light obtained with the wavelength ($\lambda$) and lipid layer thickness ($d$), and the functions $R_{\text{STDOBS}}$, $G_{\text{STDOBS}}$, $B_{\text{STDOBS}}$ of $\lambda$ represents the value of the color whose wavelength corresponds to $\lambda$ in the CIE1964 RGB standard observer. The wavelength range is from 360 to 830 nm in the visible light range provided by the CIE 1964 standard observer.

In this way, we can calculate the interference color as RGB values at different thicknesses of the meibum in the range of 0–240 nm.

## Methods

**Fig. 1:**
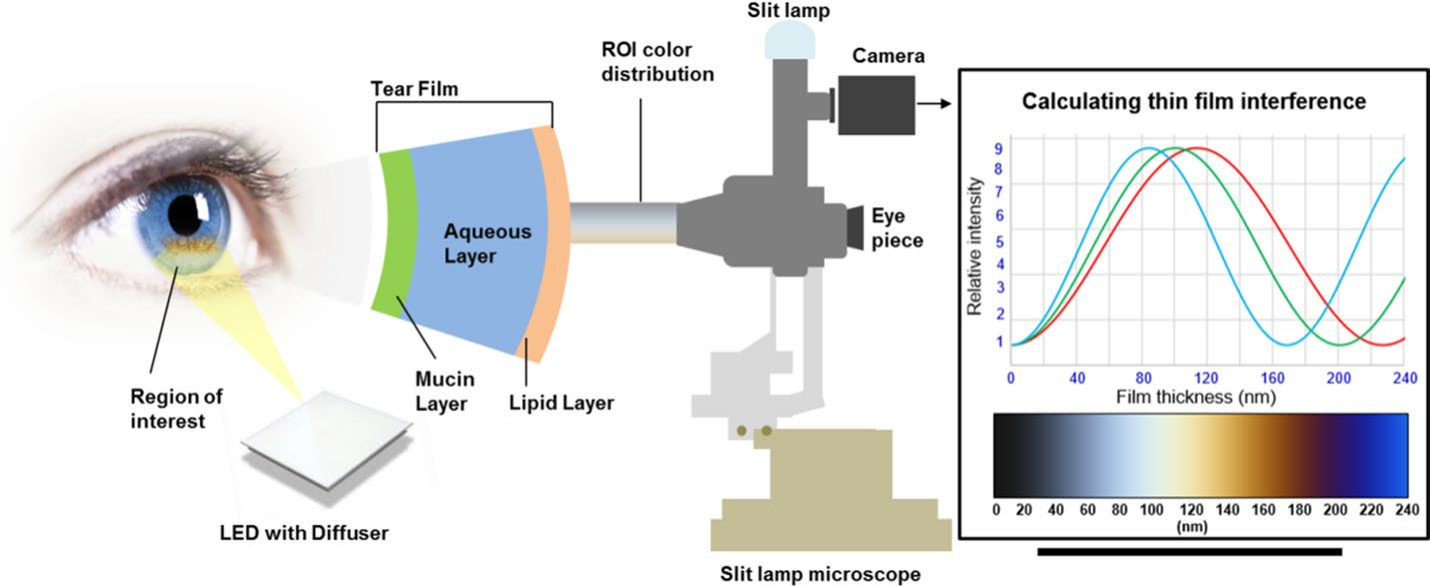
Schematic diagram of the optical system for thin film interference measurement system

We developed our algorithm to measure the lipid layer thickness quantitatively by analyzing the lipid layer interference patterns on tear film with the Lipiscanner 1.0 which consists of a LED panel (115 mm × 58 mm) covered with a polycarbonate diffuser for homogeneous illumination (Fig. 1). This simple device was used to observe the lipid layer of the tear film with a slit lamp microscope for ophthalmology and a scientific complementary metal-oxide semiconductor (sCMOS) camera. A patient’s head is placed in a fixed position on the head-chin rest of the slit lamp microscope and white light from the LED is irradiated onto the lipid layer of the eye. The white LEDs provide a color temperature of ~ 6500 K. The color from the white light interference is used to assigning the LLTs to ROI image pixels. The measurements presented in this paper represent the LLT of the precorneal tear film in the inferior iris region which was illuminated by white light.

### Instrument setup

We used the Lipiscanner 1.0 to visualize the lipid layer by white light interference, and the captured videos were used for measuring the lipid layer thickness (Fig. 1). In this setup, the LED light was illuminated onto the inferior cornea of the patients, which was then reflected onto the slit lamp microscope. The illuminating beam was not focused to a point but was spread over parts of the cornea by a diffuser. An sCMOS camera (Guppy Pro F-503, Allied Vision) was used to record the video of the interference pattern of lipid layer within the cornea region.

### Patients with dry eye syndrome

This study followed the tenets of the Declaration of Helsinki and was approved by the Institutional Review Board of Chuncheon Sacred Heart Hospital. Twenty patients with dry eyes and fourteen healthy volunteers were included in this pilot study: six patients with hyposecretory MGD (low delivery of meibum) (patient group I), seven patients with dry eye syndrome without MGD (patient group II), seven patients with hypersecretory MGD (high delivery of meibum) (patient group III), and fourteen healthy volunteers as a control group (patient group IV). An ophthalmologist who is also a meibomian gland expert (Dr. Ho Sik Hwang) categorized the patients into groups I, II and III after evaluating their medical histories [26], slit lamp examinations to measure tear break-up time [27], corneal stain, lid margin abnormality, meibum volume and quantity, Schirmer’s test [28] for tear production measurement, and meibography for morphological evaluation of the meibomian gland.

### Image processing

One of the main challenges in the processing of the eye images in our setup was that the pupil often moves suddenly during the image acquisition. To address this, we developed an algorithm that was robust even when the pupil location changed. For each patient, our algorithm processed an image sequence of 2.5 s duration (a subset of the original sequence, i.e. 75 images), and we discarded images where the pupil was not visible or was partially occluded (e.g. caused by blinking). Afterward, we started with the darkest spot in the image (which is a point in the pupil) and performed a region-growing process to extract the pupil region. We repeated this process with a different starting point to extract the iris region, from which we could extract our region of interest (ROI) of where the interferometry colors existed. Finally, we compensated colors that could be altered by the illumination light or the ambient room light so that accurate measurements of the lipid layer thickness could be achieved.

**Fig. 2:**
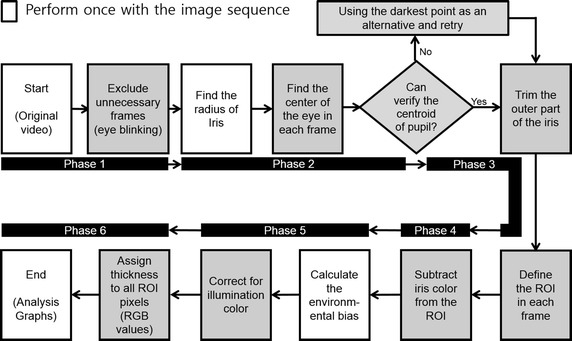
Flowchart showing the algorithm for the measurement of the lipid layer thickness

The entire image processing part of the algorithm to measure the thickness of the lipid layer runs through six phases, as shown in Fig. 2.

The Additional file 1: Movie S1 shows the procedure of image processing algorithm.

### Phase 1: Exclude unnecessary frames

First, we have to select the image frames in the raw video for analysis and discard frames that are not suitable for use. The resulting extracted video must satisfy the following conditions: The center of the pupil in each frame must be near the center of the screen (to increase recognition accuracy), and there should be no change in image brightness and zoom level between image frames.

We achieve this by filtering out frames that have no ROI, including cases when the eye closes. When eye blinking occurs, the light from the LED is strongly reflected from the eyelid, and the entire image becomes brighter. We define ${\text{B}}_{\text{opened}}$ as the brightness when the ROI is visible, and ${\text{B}}_{\text{closed}}$ as the brightness when the eye closes. Then the relationship of the values is ${\text{B}}_{\text{opened}} < \;{\text{B}}_{\text{closed}}$, where ${\text{B}}_{\text{opened}}$ is set to the mode of full frame brightness data, and ${\text{B}}_{\text{closed}}$ is set to the highest value of all brightness data. The threshold value to filter out frames including eye blinking is ${\text{B}}_{\text{opened}} \; + \;0.33\left( {{\text{B}}_{\text{closed}} \; - \;{\text{B}}_{\text{opened}} } \right)$. The coefficient of 0.33 helps to filter out the unnecessary frames and prevents false positive frames from being included in the ROI data (see Additional file 5: Image S1).

### Phase 2: Find the pupil and iris regions

Under our current image acquisition conditions, the darkest region (21 × 21 pixels) of an image in our sequence is located inside the pupil that can be used to demarcate the pupil region. Starting with the centroid of the darkest region in the image, we apply the flood-fill algorithm with 8-directions to select all pixels that belong to the pupil [29]. The Flood-fill algorithm is an algorithm that starts at a point and selects a connected group of pixels where the color (or brightness) difference between the examined pixel and the starting pixel is smaller than some selected threshold value (see Additional file 6: Image S2). Applying the flood-fill algorithm for extracting the pupil, the boundaries of the region may not be smooth and contain a portion of the iris. Thus, it is necessary to remove unwanted iris region as it affects the centroid by blurring the image followed by re-applying flood-fill algorithm with different threshold value (see Additional file 7: Image S3).

Once the image has been blurred, additional flood-fill algorithm with a lower threshold value can be used to capture the shape of the pupil with smooth boundaries without including the iris. After the pupil region is successfully extracted, the centroid of this region can be calculated which gives an approximation to the location of the center of the eye. If the location of the center of the eye obtained after correction using blurring image and the flood-fill algorithm is far from the darkest region in the pupil, we take the centroid of the darkest region as the center of the eye instead while we may partially lose the ROI.

To extract the iris region, we apply the same flood-fill algorithm again with a starting point outside the pupil (i.e. a point in the iris). We then perform the Canny edge detection [30] on the resulting region to extract the boundary pixels. However, as the iris is partially covered by the eyelid most of the time, the resulting boundary consists of many false boundaries of the iris. To overcome this, we examine only the boundary pixels that have almost vertical edge orientations and are within an empirically pre-defined distance from the detected center of the eye (in our data, the diameter of iris was approximately 480 pixels. However, this number depends on the camera zoom state and patient’s eye trait. Thus, in practice this iris size has to be determined using the iris from image.) It is because in order to get as much ROI as possible, we need the exact radius of the iris but already know the approximate value. From these boundary pixels, we can take their average distances from the center of the eye and estimate the radius of the iris.

### Phase 3: Extract the region of interest

**Fig. 3:**
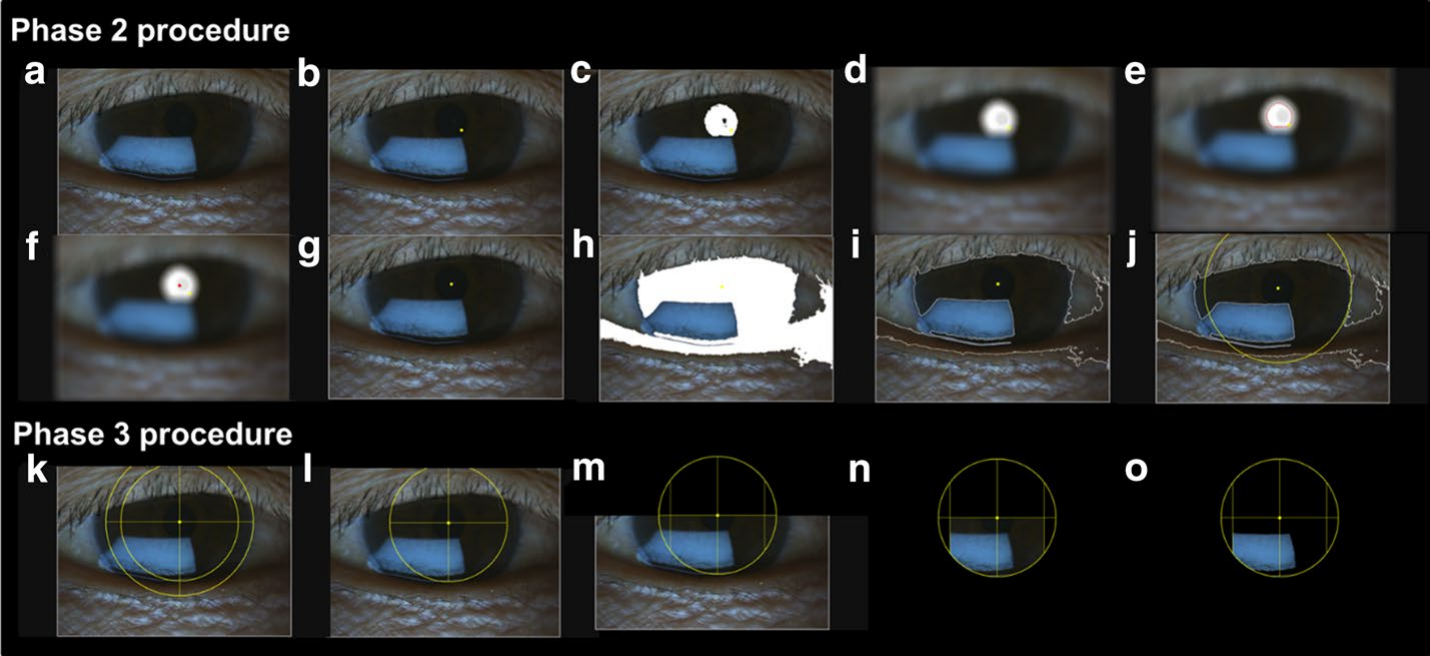
Detailed image processing steps for automatic ROI tracking and background subtraction (Phase 2–3). **a–e** Finding the pupil (Phase 2). **f–j** Finding the iris (Phase 2). **k–o** Extracting the ROI (Phase 3) (see Additional file 1: Movie S1)

The ROI should be the region within the iris that shows interference colors that should appear in the bottom half of the iris image. Thus, we choose our ROI to the area within 80% of the measured iris to prevent the lower eyelid from being included in the ROI and to exclude the sclera as part of ROI in case the centroid of pupil is inaccurate. We then crop away the regions above the center of the circle, the regions outside of the circle, and for the region within the circle, we also crop away small regions on the left and right that are farther than 80% of the radius of the new inner circle. This is to prevent the appearance of saturated pixels from the white sclera of the eye. After that, pixels that have a brightness less than the average brightness of the region are also removed. The resulting region is our ROI. Figure 3 shows some of the intermediate images in phases 2 and 3 and the final ROI.

### Phase 4: Subtract iris color from region of interest

The colors in the ROI originate from a combination of the white light interference and the iris color. We need to subtract the iris color component from the ROI, or otherwise, it can affect the subsequent color analysis. We achieve this by finding the color of the iris in the phase 3 circle outside of the ROI, and then subtract it from the ROI.

### Phase 5: Correct for illumination colors

Depending on the color and brightness of the room lighting or camera exposure value at the time of image acquisition, the colors in the image may appear to be different from the actual ones. Also, the color temperature of the white light that we used to calculate the theoretical color corresponding to the thickness is different from that of the LED light of the Lipiscanner. To compensate for this, we estimated how much the RGB values were biased from the white color of the sclera of the patient’s eye captured under the same conditions and applied the corresponding correction to the ROI.

### Phase 6: Assign thickness to all ROI pixels

We map each pixel’s color to a thickness value of the lipid layer using a lookup table represented by the three-dimensional solid curve in the RGB space (Fig. 6). The lookup table is obtained by applying the Fresnel equations to the reflection-transmission model and then plotting the color output against a different lipid film thickness input. Color values that do not match any values in the lookup table are approximated by the color in the lookup table that has the closest Euclidean distance from it. In this way, the distribution of LLT variation within each frame and across image frames can be assessed, and the mean and standard deviation of LLT can be calculated.

## Results

### Region of interest tracking

The automated ROI tracking technique and some intermediate results from Phase 2 and 3 of our algorithm are shown in Fig. 3 with one exemplary image frame.

- Preparing original images from a video clip (Fig. 3a).
- Searching for the darkest point in the image (the white dot inside the pupil) (Fig. 3b).
- Identifying of the pupil region of the eye (white color) (Fig. 3c).
- Blurring of the image (Fig. 3d).
- Determining the pupil of the eye (marked with red boundary) (Fig. 3e).
- Locating the centroid of the pupil (the red dot) (Fig. 3f).
- Calculating the centroid (the white dot) (Fig. 3g).
- Searching for the iris region (white area) (Fig. 3h).
- Finding the boundary of the iris region (Fig. 3i).
- Determining the radius of the iris and drawing a circle (Fig. 3j).
- Defining a smaller subset region of the iris (Fig. 3k).
- Setting the radius of a smaller circle (Fig. 3l).
- Cropping the area above the center (Fig. 3m).
- Selecting the area inside of the small circle (Fig. 3n).

Setting the region of interest (Fig. 3o).

### Statistical analysis of lipid layer thickness over time

**Fig. 4:**
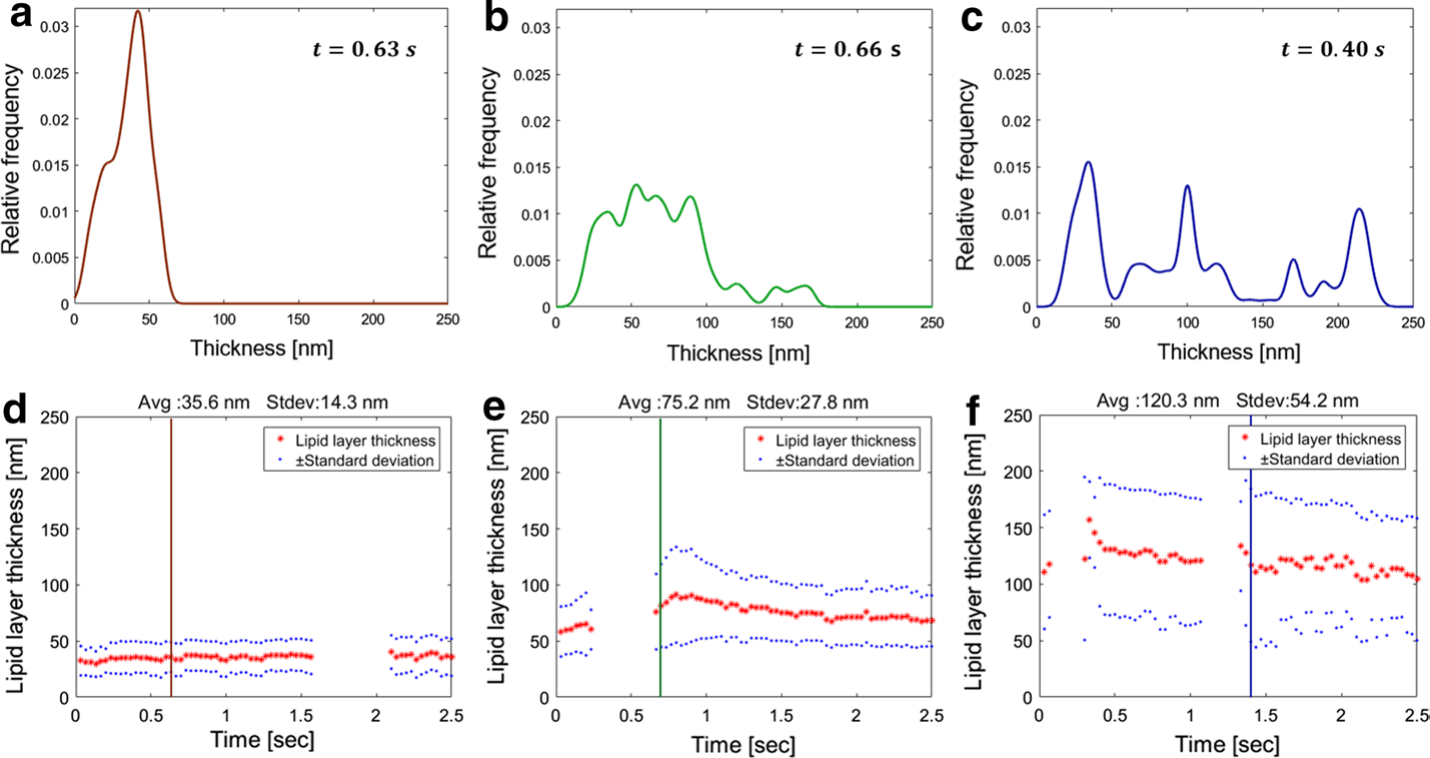
Statistical information of lipid layer thickness over time. **a**–**c** relative frequency distribution of each patient’s LLT at selected time points of 0.63, 0.66, and 1.4 s, respectively. **d**–**f** per-frame average LLT and standard deviation over the entire image sequence. Selected time points for (**a**–**c**) are marked with vertical lines. Patient I has hyposecretory MGD (**a**, **d**), patient II has dry eye without MGD (**b**, **e**), and patient III has hypersecretory MGD (**c**, **f**) (see Additional files 5, 6, 7)

The quantitative measurements of the lipid layer thickness from three representative patients in each group are summarized in Fig. 4. Patient I has hyposecretory MGD, patient II has dry eye without MGD, and patient III has hypersecretory MGD. The relative frequency distributions of the lipid layer thickness at selected time points in the three patients are shown in Fig. 4a–c. The average lipid layer thickness and the standard deviation per frame are also computed and plotted for the entire image sequence. The mean lipid layer thicknesses for patient I, II and III were measured as 35.6 ± 14.3, 75.2 ± 27.8, 120.3 ± 54.2 nm (average ± standard deviation), respectively from the data in Fig. 4d–f. A real-time display of the analysis of the lipid layer thickness for the three patients including means, standard deviations, and a relative frequency graph for each frame are included in the Additional files (see Additional file 2: Movies S2, Additional file 3: Movie S3, Additional file 4: Movie S4).

### Visualization of lipid layer thickness information in space

**Fig. 5:**
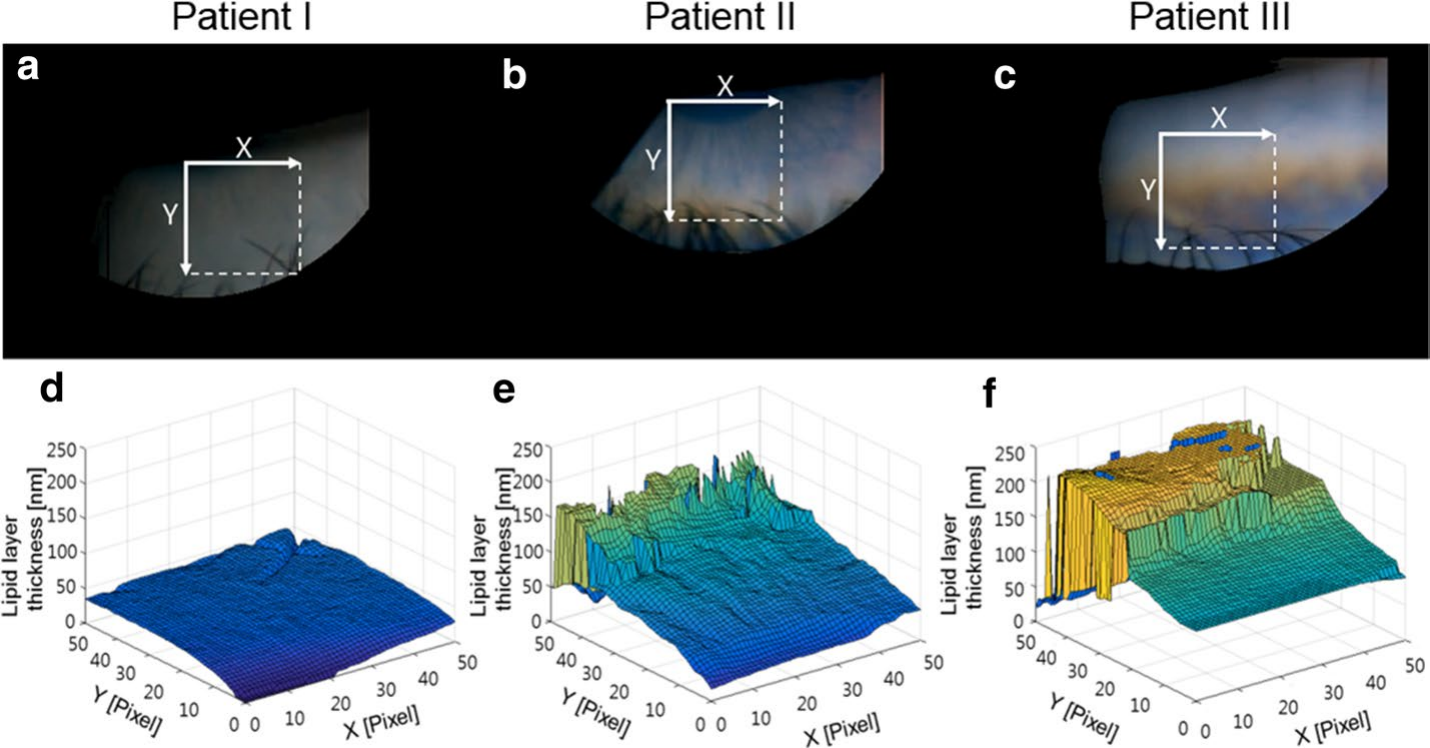
Spatial visualization of lipid layer thickness distribution. **a**–**c** The ROI region for each patient at a selected frame **d**–**f** 3D plots displaying each patient’s lipid layer thickness in a 50 × 50-pixel grid (i.e. 2500 pixels). The entire ROI contains approximately 5000–7000 pixels. Patient I has hyposecretory MGD (**a**, **d**), patient II has dry eye without MGD (**b**, **e**), and patient III has hypersecretory MGD (**c**, **f**)

We constructed a three-dimensional (3D) visualization of the structure of each patient’s lipid layer for individual assessment of the lipid layer thickness of the patients’ data shown in Fig. 5. This visualization with color coding for the lipid layer thickness can help us to examine overall LLT distribution and parts that are exceptionally thin or thick. The closer the ROI is to the lower eyelid, the thicker the LLT (the direction in which y increases). Due to the eyelash shadows on the ROI, we have a pixel that does not show a uniform LLT on the 3D surface plots. The LLT value in eyelash shadows is close to zero and is not included in the analysis.

### LLT assignment of image pixel values in RGB 3D space

**Fig. 6:**
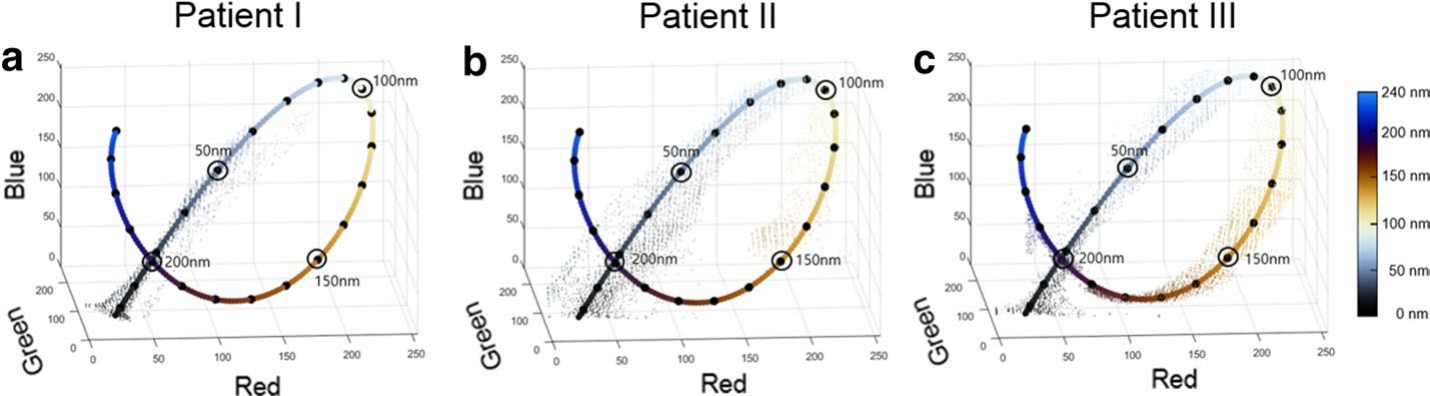
Scatter plot of LLT data distribution in RGB 3D space from representative patients. **a** Patient with hyposecretory MGD (**b**) Patient without MGD (**c**) Patient with hypersecretory MGD. The color scale curve represents theoretical LLT matching curve in the RGB space while each dot represents the real pixel data’s RGB value. All of the dots are assigned corresponding colors within LLT lookup table. The black dots overlaying the solid line mark an interval of 10 nm

Figure 6 shows plots of the compensated image pixel’s color data from the three patients. The color scale curve—3D representation of color bar on the right side—represents theoretical LLT values in three-dimensional RGB space. The individual dots of scatter plot represent the image pixel’s RGB color data, and the color of each data represents the assignment of LLT based on the closest Euclidean distance from the color lookup table. Patient I image pixel data is distributed around the low LLT, Patient II data is near 100 nm, and Patient III data is distributed near the high LLT graph. The number of image data pixels in a graph is about 3000–5000. Data were obtained from three image frames, including the front and back frames of the image frame used to draw the 3D surface plots (Fig. 5). We downsized the number of data in the graph to one-third and used a scatter plot.

### Comparison of the LLT and clinical data of patients with dry eye syndrome

**Fig. 7:**
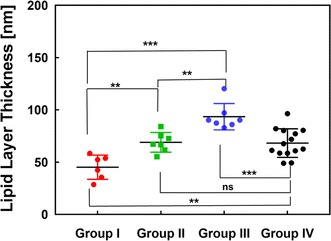
A Comparison of lipid layer thickness (LLT) between four different groups. Group I (Hyposecretory MGD patients), Group II (dry eye syndrome without MGD), Group III (hypersecretory MGD patients), and Group IV (healthy volunteers as a control group). (n = 6, 7, 7, 14 patients for Group I, II, III, IV respectively), **indicates p < 0.01, ns indicates p > 0.5 and ***indicates p < 0.001

As a proof-of-principle, we used a total of thirty-four patients with dry eye syndrome for this feasibility study. Their data were blindly analyzed with our image processing algorithm without any clinical information of the patients. Based on routine clinical examinations, an ophthalmologist categorized the patients into four groups: hyposecretory MGD (low delivery of meibum, the patient group I), dry eye syndrome without MGD (patient group II), hypersecretory MGD (high delivery of meibum, patient group III), and Group IV (healthy volunteers as a control group). The results of the LLT measurements for each category is compared in Fig. 7. Because changes in the LLT as captured in the patient images are slow and continuous, using a camera system at 30 fps was sufficient to obtain the results. With the limited data set, the LLT of hyposecretory MGD was thinner (45.2 ± 11.6 nm) than that of dry eye syndrome without MGD (69.0 ± 9.43 nm), and the LLT of hypersecretory MGD was thicker (93.5 ± 12.4 nm) than that of dry eye syndrome without MGD and healthy volunteers (68.3 ± 13.7 nm). There were statistical differences between the three groups (a one-way ANOVA).

## Discussion

The purpose of this study was to develop an image-based algorithm for quantitative analysis of tear film lipid layer thickness (see Additional file 8: Image S8). Our algorithm was robust enough to work despite fluctuations and eye movements during the video recording. For example, the ambient room light did not affect our measurement as the illumination was relatively bright. In addition, we used a low-grade scientific camera with relatively high noise that has not been problematic.

We created a video clip to explain our algorithm involving the steps and results (see Additional file 1: Movie S1). We analyzed the ROI on the dark iris in Fig. 3o of phase 2 because we performed the image processing with Asian patients with dark irises. When the same algorithm was used on a bright-colored iris, the algorithm might have limitation identifying ROI.

We subtracted the RGB average value of iris from the ROI to remove the iris color within the ROI region. However, this approach may not eliminate the noise generated by the iris pattern. To effectively remove iris background, we may obtain the iris data under the ROI separately and subtract the iris value for each pixel (Fig. 2 Phase 4). We used the sclera of the patient’s eye for the white color reference. In future analysis, this color variation may be adjusted by using an image or video taken under the same recording conditions using a white paper (i.e., the fiducial marker for color) under the LED light. As an additional method, HSV color space can be adopted to iris color detection phase, which is considered a more effective color space than RGB color space for iris authentication [31]. This approaches would produce more consistent results than using potentially non-identical sclera color from each patient (Fig. 2 Phase 5).

So far, algorithms have been serialized using only a single core from a quadcore CPU (Intel i7-6700), so it is relatively slow and currently takes approximately 3 min (1.5 s/frame) to determine the radius and compensate image color with a video clip. We expect to achieve comparable results in less than a minute by using a graphic processing unit (GPU) with parallel processing for executing the algorithm shown in Fig. 2.

Color bar values from the theoretical calculation are similar to the patient’s LLT values derived by the newly developed algorithm. While only limited number of patients’ data were available for our pilot study, the results were reasonable based on the previous study that demonstrated the correlation between the lipid layer thickness and two frequently used dry eye tests by comparing results of tear break-up time and Schirmer’s test [32]. Therefore, our approach can be useful in providing objective information to evaluate the lipid layer thickness for patients with dry eye syndrome. For further verification, we are currently analyzing more patients with our algorithm along with their clinical manifestation.

## Conclusion

In this study, we utilized white light interference from the lipid layer and implemented a novel algorithm to analyze the thickness of the lipid layer by examining interference color distribution. Our proposed algorithm provides a quantitative measure of the lipid layer thickness even in the presence of eye movement by tracking ROI for each frame. With a small number of patient data set as a feasibility study, our experimental results demonstrate that the lipid layer thickness was different in the subcategories of dry eye patients evaluated by an ophthalmologist. This approach provides a significant step forward in developing a fully automated instrument for evaluation of dry eye category and thus guiding optimal treatment for patients.

## Additional files

**Additional file 1.** The progress of image processing from phase 1 to 6.

**Additional file 2.** Real-time change of lipid layer thickness in hyposecretory MGD (low delivery of meibum) (patient I).

**Additional file 3.** Real-time change of lipid layer thickness without MGD (Dry eye syndrome) (patient II).

**Additional file 4.** Real-time change of lipid layer thickness in hypersecretory MGD (high delivery of meibum) (patient III).

**Additional file 5.** A figure that shows how to filter out frames that include eye-closing motion or closed eye. We set the value to less than the average between ${\text{B}}_{\text{opened}}$ and ${\text{B}}_{\text{closed}}$ (threshold = 0.5) to prevent the transitioning frames be regarded as falsely opened state (ROI data). On the other hand, we had to set the threshold higher than 0.25 which was the variation of ROI brightness. Thus, we set our threshold as 0.33 which provided reasonable results most of the time (98% of the case).

**Additional file 6.** The relationship between the threshold value and the captured shape of the pupil. The threshold value to identify pupil in our flood-fill algorithm was chosen as 5 out of 256 (8 bit data type.) (i) If we choose a lower threshold, only central sub-region of the pupil could be recognized as a full pupil (see threshold = 3 cases.) (ii) If we choose a higher threshold, then some regions of the iris could be incorrectly recognized as the pupil (see threshold = 7 cases).

**Additional file 7.** Schematic diagram of additional steps to refine the pupil. With the original flood-fill algorithm was applied, the algorithm could misinterpret a portion of the iris as a pupil in some cases (Fig. S4a) In order to remove the mistaken region for iris, we applied a white color (brightness = 255) to the region recognized as a pupil (Fig. S4b,) blurred the image (Fig. S4c,) and then applied additional flood-fill algorithm with a lower threshold value (Fig. S4d, 3 in our case.) This value gave satisfactory results in most of the cases (Fig. S4e, > 99.9%).

**Additional file 8.** Screenshot of an analysis movie combined with original video, processed video, and lipid layer thickness data distribution. The top left corner of the video shows the original video, the top right shows the processed video, the bottom left shows the thickness data distribution for each frame, and the bottom right shows the summary.

## Abbreviations

ANOVA: analysis of variance
CIE: Commission International de I’Eclairage
CPU: central processing unit
DES: dry eye syndrome
GPU: graphics processing unit
HSV: Hue-saturation-value
INT: interference
LED: light emitting diode
LLT: lipid layer thickness
MGD: meibomian gland dysfunction
OPD: optical path difference
RGB: red–green–blue
ROI: region of interest
sCMOS: scientific complementary metal-oxide semiconductor
STDOBS: standard observer

## References

[1] Lemp MA (2008). Advances in understanding and managing dry eye disease. Am J Ophthalmol.

[2] Lemp A (1995). Report of the National Eye Institute/Industry Workshop on clinical trials in dry eyes. Eye Contact Lens.

[3] Sullivan DA, Sullivan BD, Evans JE, Schirra F, Yamagami H, Liu M (2002). Androgen deficiency, meibomian gland dysfunction, and evaporative dry eye. Ann N.Y. Acad Sci.

[4] Driver PJ, Lemp MA (1996). Meibomian gland dysfunction. Surv Ophthalmol.

[5] Knop E, Knop N, Millar T, Obata H, Sullivan DA (2011). The international workshop on meibomian gland dysfunction: report of the subcommittee on anatomy, physiology, and pathophysiology of the meibomian gland. Invest Ophthalmol Vis Sci.

[6] McCulley JP, Shine WE (2004). The lipid layer of tears: dependent on meibomian gland function. Exp Eye Res.

[7] McCulley JP, Shine WE (2003). Meibomian gland function and the tear lipid layer. Ocular Surf.

[8] Foulks GN (2007). The correlation between the tear film lipid layer and dry eye disease. Surv Ophthalmol.

[9] Eom Y, Lee J-S, Kang S-Y, Kim HM, Song J-S (2013). Correlation between quantitative measurements of tear film lipid layer thickness and meibomian gland loss in patients with obstructive meibomian gland dysfunction and normal controls. Am J Ophthalmol.

[10] Blackie CA, Solomon JD, Scaffidi RC, Greiner JV, Lemp MA, Korb DR (2009). The relationship between dry eye symptoms and lipid layer thickness. Cornea.

[11] Savini G, Prabhawasat P, Kojima T, Grueterich M, Espana E, Goto E (2008). The challenge of dry eye diagnosis. Clin Ophthalmol.

[12] Goto E, Tseng SC (2003). Differentiation of lipid tear deficiency dry eye by kinetic analysis of tear interference images. Arch Ophthalmol.

[13] Bai Y, Nichols JJ (2017). Advances in thickness measurements and dynamic visualization of the tear film using non-invasive optical approaches. Prog Retin Eye Res.

[14] McDonald JE (1968). Surface phenomena of tear films. Trans Am Ophthalmol Soc.

[15] Hamano H, Hori M, Kawabe H, Umeno M, Mitsunaga S, Ohnishi Y, Koma I (1980). Clinical applications of bio differential interference microscope. Eye Contact Lens.

[16] Guillon J-P (1982). Tear film photography and contact lens wear. J Br Contact Lens Assoc.

[17] Doane MG, Lee ME (1998). Tear film interferometry as a diagnostic tool for evaluating normal and dry-eye tear film. Adv Exp Med Biol.

[18] Khamene A, Negahdaripour S, Tseng SC (2000). A spectral-discrimination method for tear-film lipid-layer thickness estimation from fringe pattern images. IEEE Trans Biomed Eng.

[19] Hwang HS, Kim EC, Kim MS (2014). Novel tear interferometer made of paper for lipid layer evaluation. Cornea.

[20] Yokoi N, Takehisa Y, Kinoshita S (1996). Correlation of tear lipid layer interference patterns with the diagnosis and severity of dry eye. Am J Ophthalmol.

[21] Goto E, Tseng SC (2003). Kinetic analysis of tear interference images in aqueous tear deficiency dry eye before and after punctal occlusion. Invest Ophthalmol Vis Sci.

[22] Borrego L, Saenz-Frances F, Finis D, Benitez-del-Castillo J, Geerling G (2013). Role of lipid emulsion eye drops in the improvement of lipid layer thickness measured with Lipiview. Invest Ophthalmol Vis Sci.

[23] Yokoi N, Komuro A (2004). Non-invasive methods of assessing the tear film. Exp Eye Res.

[24] Carlson AN. A new paradigm for treating dry eye patients. Adv Ocular Care [Review]. 2010:37-41.

[25] Hapke B (1981). Bidirectional reflectance spectroscopy: 1. Theory. J Geophys Res Solid Earth.

[26] Doughty MJ, Fonn D, Richter D, Simpson T, Caffery B, Gordon K (1997). A patient questionnaire approach to estimating the prevalence of dry eye symptoms in patients presenting to optometric practices across Canada. Optom Vis Sci.

[27] Lemp MA, Hamill JR (1973). Factors affecting tear film breakup in normal eyes. Arch Ophthalmol.

[28] Lamberts DW, Foster CS, Perry HD (1979). Schirmer test after topical anesthesia and the tear meniscus height in normal eyes. Arch Ophthalmol.

[29] Arvo J, Hirvikorpi M, Tyystjärvi J (2004). Approximate soft shadows win an image-space flood-fill algorithm. Computer Gr Forum.

[30] Canny J (1986). A computational approach to edge detection. IEEE Trans Pattern Anal Mach Intell.

[31] Dantcheva A, Erdogmus N, Dugelay J-L. On the reliability of eye color as a soft biometric trait. In: Application of computer vision (WACV). 2011. p. 227–31.

[32] Isreb M, Greiner J, Korb D, Glonek T, Mody S, Finnemore V (2003). Correlation of lipid layer thickness measurements with fluorescein tear film break-up time and Schirmer’s test. Eye.

