# Supplementary figures and images for: Image-based quantitative analysis of tear film lipid layer thickness for meibomian gland evaluation
Source: Biomed Eng Online. 2017 Nov 23;16:135. doi: 10.1186/s12938-017-0426-8 (PMC5701431; doi:10.1186/s12938-017-0426-8)

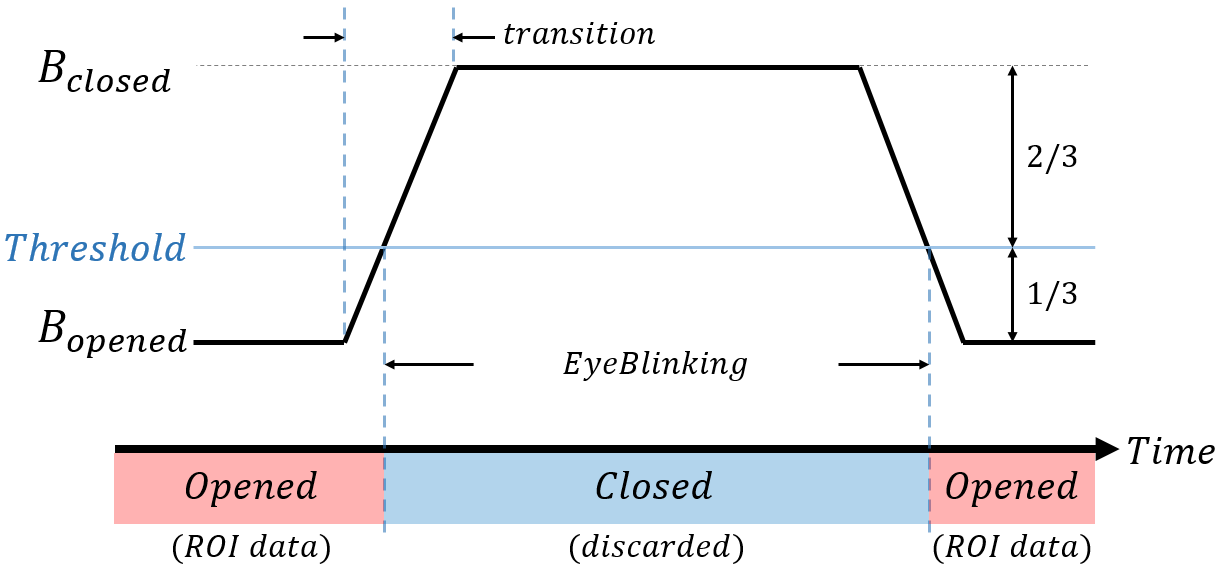

Supplement: Supplementary file 5 — Additional file 5. A figure that shows how to filter out frames that include eye-closing motion or closed eye. We set the value to less than the average between ${\text{B}}_{\text{opened}}$Bopened and ${\text{B}}_{\text{closed}}$Bclosed (threshold = 0.5) to prevent the transitioning frames be regarded as falsely opened state (ROI data). On the other hand, we had to set the threshold higher than 0.25 which was the variation of ROI brightness. Thus, we set our threshold as 0.33 which provided reasonable results most of the time (98% of the case). [file 12938_2017_426_MOESM5_ESM.png]

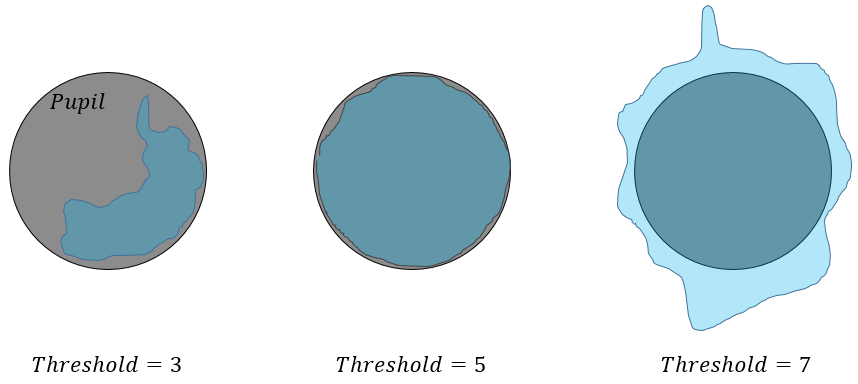

Supplement: Supplementary file 6 — Additional file 6. The relationship between the threshold value and the captured shape of the pupil. The threshold value to identify pupil in our flood-fill algorithm was chosen as 5 out of 256 (8 bit data type.) (i) If we choose a lower threshold, only central sub-region of the pupil could be recognized as a full pupil (see threshold = 3 cases.) (ii) If we choose a higher threshold, then some regions of the iris could be incorrectly recognized as the pupil (see threshold = 7 cases). [file 12938_2017_426_MOESM6_ESM.png]

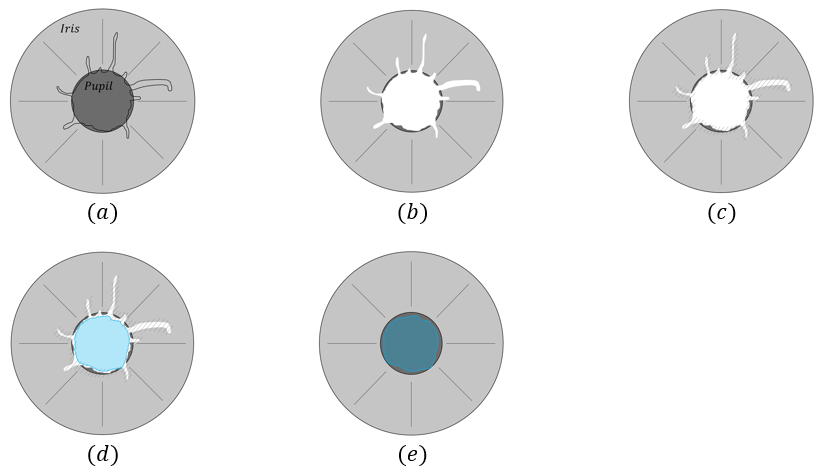

Supplement: Supplementary file 7 — Additional file 7. Schematic diagram of additional steps to refine the pupil. With the original flood-fill algorithm was applied, the algorithm could misinterpret a portion of the iris as a pupil in some cases (Fig. S4a) In order to remove the mistaken region for iris, we applied a white color (brightness = 255) to the region recognized as a pupil (Fig. S4b,) blurred the image (Fig. S4c,) and then applied additional flood-fill algorithm with a lower threshold value (Fig. S4d, 3 in our case.) This value gave satisfactory results in most of the cases (Fig. S4e, > 99.9%). [file 12938_2017_426_MOESM7_ESM.png]

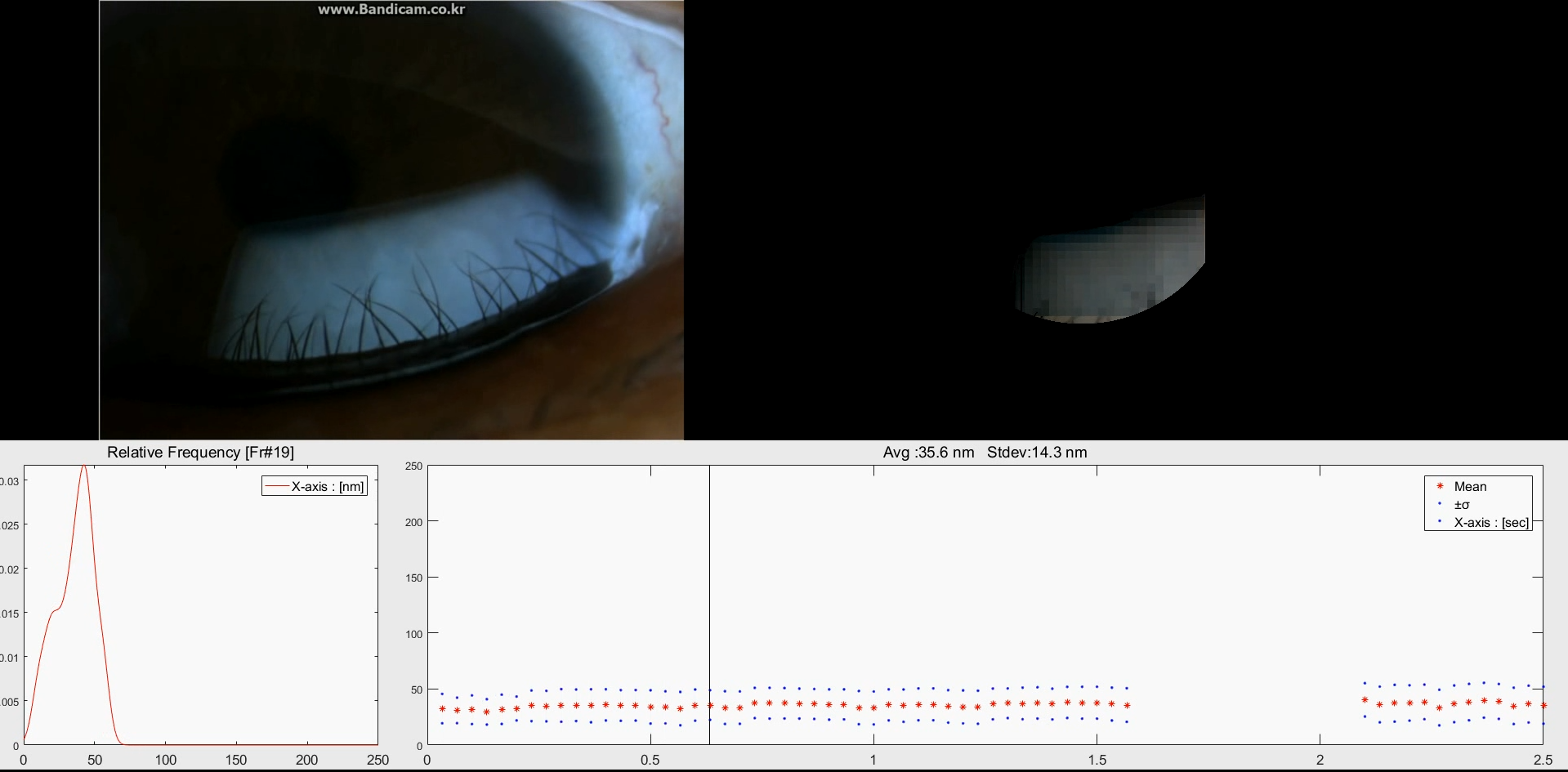

Supplement: Supplementary file 8 — Additional file 8. Screenshot of an analysis movie combined with original video, processed video, and lipid layer thickness data distribution. The top left corner of the video shows the original video, the top right shows the processed video, the bottom left shows the thickness data distribution for each frame, and the bottom right shows the summary. [file 12938_2017_426_MOESM8_ESM.png]
